# Top-down GaN nanowire transistors with nearly zero gate hysteresis for parallel vertical electronics

**DOI:** 10.1038/s41598-019-46186-9

**Published:** 2019-07-16

**Authors:** Muhammad Fahlesa Fatahilah, Feng Yu, Klaas Strempel, Friedhard Römer, Dario Maradan, Matteo Meneghini, Andrey Bakin, Frank Hohls, Hans Werner Schumacher, Bernd Witzigmann, Andreas Waag, Hutomo Suryo Wasisto

**Affiliations:** 1 Institute of Semiconductor Technology (IHT), Technische Universität Braunschweig, Hans-Sommer-Straße 66, D-38106 Braunschweig, Germany; 2 Laboratory for Emerging Nanometrology (LENA), Technische Universität Braunschweig, Langer Kamp 6, D-38106 Braunschweig, Germany; 3 Physikalisch-Technische Bundesanstalt (PTB), Bundesallee 100, D-38116 Braunschweig, Germany; 4 Computational Electronics and Photonics (CEP), University of Kassel, Wilhelmshöher Allee 71, D-34121 Kassel, Germany; 5 Department of Information Engineering, University of Padua, 35131 Padua, Italy

**Keywords:** Electrical and electronic engineering, Electronic and spintronic devices

## Abstract

This paper reports on the direct qualitative and quantitative performance comparisons of the field-effect transistors (FETs) based on vertical gallium nitride nanowires (GaN NWs) with different NW numbers (i.e., 1–100) and diameters (i.e., 220–640 nm) fabricated on the same wafer substrate to prove the feasibility of employing the vertical 3D architecture concept towards massively parallel electronic integration, particularly for logic circuitry and metrological applications. A top-down approach combining both inductively coupled plasma dry reactive ion etching (ICP-DRIE) and wet chemical etching is applied in the realization of vertically aligned GaN NWs on metalorganic vapor-phase epitaxy (MOVPE)-based GaN thin films with specific doping profiles. The FETs are fabricated involving a stack of *n-p-n* GaN layers with embedded inverted *p*-channel, top drain bridging contact, and wrap-around gating technology. From the electrical characterization of the integrated NWs, a threshold voltage (*V*_th_) of (6.6 ± 0.3) V is obtained, which is sufficient for safely operating these devices in an enhancement mode (E-mode). Aluminium oxide (Al_2_O_3_) grown by atomic layer deposition (ALD) is used as the gate dielectric material resulting in nearly-zero gate hysteresis (i.e., forward and backward sweep *V*_th_ shift (Δ*V*_th_) of ~0.2 V). Regardless of the required device processing optimization for having better linearity profile, the upscaling capability of the devices from single NW to NW array in terms of the produced currents could already be demonstrated. Thus, the presented concept is expected to bridge the nanoworld into the macroscopic world, and subsequently paves the way to the realization of innovative large-scale vertical GaN nanoelectronics.

## Introduction

Since the invention of the first integrated circuit (IC) in 1958, planar metal-oxide semiconductor field-effect transistors (MOSFETs) based on silicon have been dominating in the global microelectronics industry and continuously used to build electronic devices, such as modern microprocessors, which up-to-date can integrate more than one billion transistors on a single chip^1–3^. The raised challenges following this microelectronic revolution are still how to fabricate the transistors more efficiently by keeping the smallest possible footprint and how to better exploit the devices containing billions of transistors. Even though the electronic engineers and scientists have attempted to further miniaturize and to put more transistors on it as well as to improve their performance, the technological bottleneck still occurs as the available active area or footprint of the whole device at a certain point is constrained, leading to the limitation of the integrated electronic building blocks. Therefore, although the development of microelectronics and the following enhancement in circuit performance have generally been driven by the downscaling of the basic electronic component (i.e., the MOSFET), it has faced physical limitations of nanoscale transistor operation, which then leads to the research and development of more innovative MOSFET architectures, e.g., to improve electrostatic control of the channel. Thus, several novel approaches have been investigated including the usage of other semiconductor materials (e.g., silicon carbide (SiC)^4^ and gallium nitride (GaN)^5–7^ for specific applications (e.g., in high temperature and power switching devices), planar 3D nanostructures (e.g., horizontal nanowire (NW) transistors)^8,9^, and vertical 3D architectures (e.g., FinFET, NW FET, and tri-gate architectures)^10–14^.

Among others, GaN transistors provide prospects for making monolithically integrated electronics-photonics platforms because of their direct wide band gap, even though the already realized devices are still in a planar architecture^15,16^. As the figure of merits for semiconductor power devices, the high breakdown voltage (BV) and low on resistance (*R*_on_) are required, in which BV > 800 V and *R*_on_ ≈ 0.36 m Ω cm^2^ can be obtained by vertical GaN FinFET based on bulk GaN wafer^14^. In quantum engineered transistors and logic applications, this vertical method also gives a new strategy towards higher performance and integration of *p*- and *n*-channel transistors^17,18^. From our point of view, the vertical 3D architecture has become more attractive because it provides more advantages: (1) it can minimize the current collapse, thanks to the absence of surface-related trapping phenomena; (2) the gate length (*L*) is not limited by lithography process; (3) gating technology can be flexibly designed (e.g., wrap around gate); (4) vertical parallel current paths and collection can be obtained on a small footprint for high scalability; (5) better thermal performance, at which the maximum temperature is close to top part of NWs, brought potential to achieve more power density; and (6) contrary to lateral devices, where breakdown voltage scales with area (and cost), in vertical devices the breakdown voltage is only dependent on the thickness/properties of the epitaxial stacks^13,19–25^.

In the last few years, several vertical electronic devices based on semiconductor NWs (e.g., Si and GaN) have been demonstrated with different types of transistors, and only a few of them concern about the direct device scaling behavior as affected by the modified number and diameter of NWs^26–28^. The diameter size and doping concentration of *n-n-n* or *n-i-n* GaN epitaxial NWs were reported to be able to determine the operation modes of the GaN FETs (normally-off or normally-on)^29,30^. The threshold voltage can be increased by inserting a *p*-channel inside the wire structure instead of *n*-channel^13,28,30^. Nevertheless, those devices exhibited very large drain current hysteresis during bidirectional gate sweep, which is assumed to be due to the unintentionally introduced mobile ions. Meanwhile, for vertical Si FETs, the massively parallel dense NW arrays with wrap around gate structure had been reported to have an improved electrostatic control during device operation, a smaller transistor chip size, and a low leakage current^26^. However, those nanotransistors were fabricated using electron beam lithography, which definitely lowers their potential to be transferred in batch production because of the higher device processing cost and longer production time. Thus, other lithography techniques (e.g., photolithography, nanoimprint lithography, and colloidal lithography) have been currently employed to produce vertical Si NW arrays, although such production of vertical Si NW FET devices using those techniques has not been demonstrated so far^31–38^.

In this paper, a direct proof of device current scaling and parallel transistor integration is demonstrated using vertical *n-p-n* GaN NW FETs with almost-zero Δ*V*_th_ hysteresis that were fabricated using top-down approach (i.e., standard UV-photolithography and hybrid etching processes). Al_2_O_3_ thin layers fabricated by ALD process using trimethylaluminium and water were employed as gate dielectrics instead of SiO_2_ films that are commonly used for Si FETs. Owing to various patterns on the mask, the electrical characteristics of the realized 1-, 9-, and 100-NW FETs with different diameters were extracted and analyzed. In case of the top drain electrode configuration, mesa structures have been added as mechanical support for more robust electrical characterization and circuit integration (e.g., during wire bonding).

## Results and Discussion

**Figure 1:**
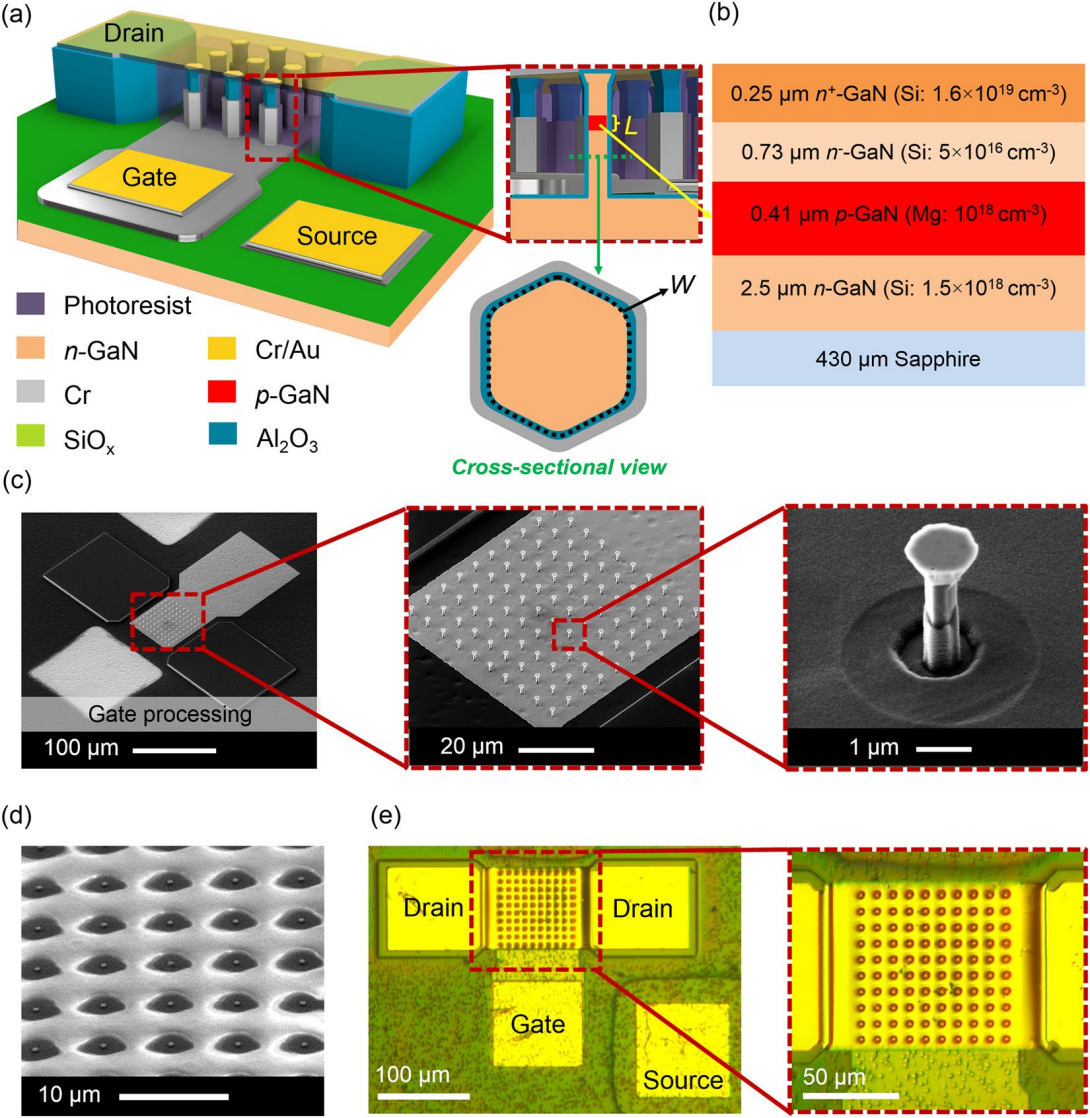
(**a**) 3D schematics of a 9 *n-p-n* vertical GaN NW FET with *L* and *W* as gate length and gate width in inset, respectively, and (**b**) wafer composition used in this work. Fabrication process steps for creating 1, 9, and 100 NW FETs after (**c**) Cr gate processing, (**d**) photoresist filling, short UV exposure, and curing for 30 min at 250 °C, (**e**) e-beam evaporation of Cr/Au (80/200 nm) and subsequent etch-back process to form metal drain contact on *n-p-n* vertical GaN NW array.

Figure 1a depicts the 3D device schematic based on the *n-p-n* GaN epi-layer provided in Fig. 1b. The doping concentration of the channel region has a significant impact on the operation mode of the transistors, which influences the threshold voltage. In our devices, the channel is *p*-doped to obtain sufficiently high threshold voltages.

**Figure 2:**
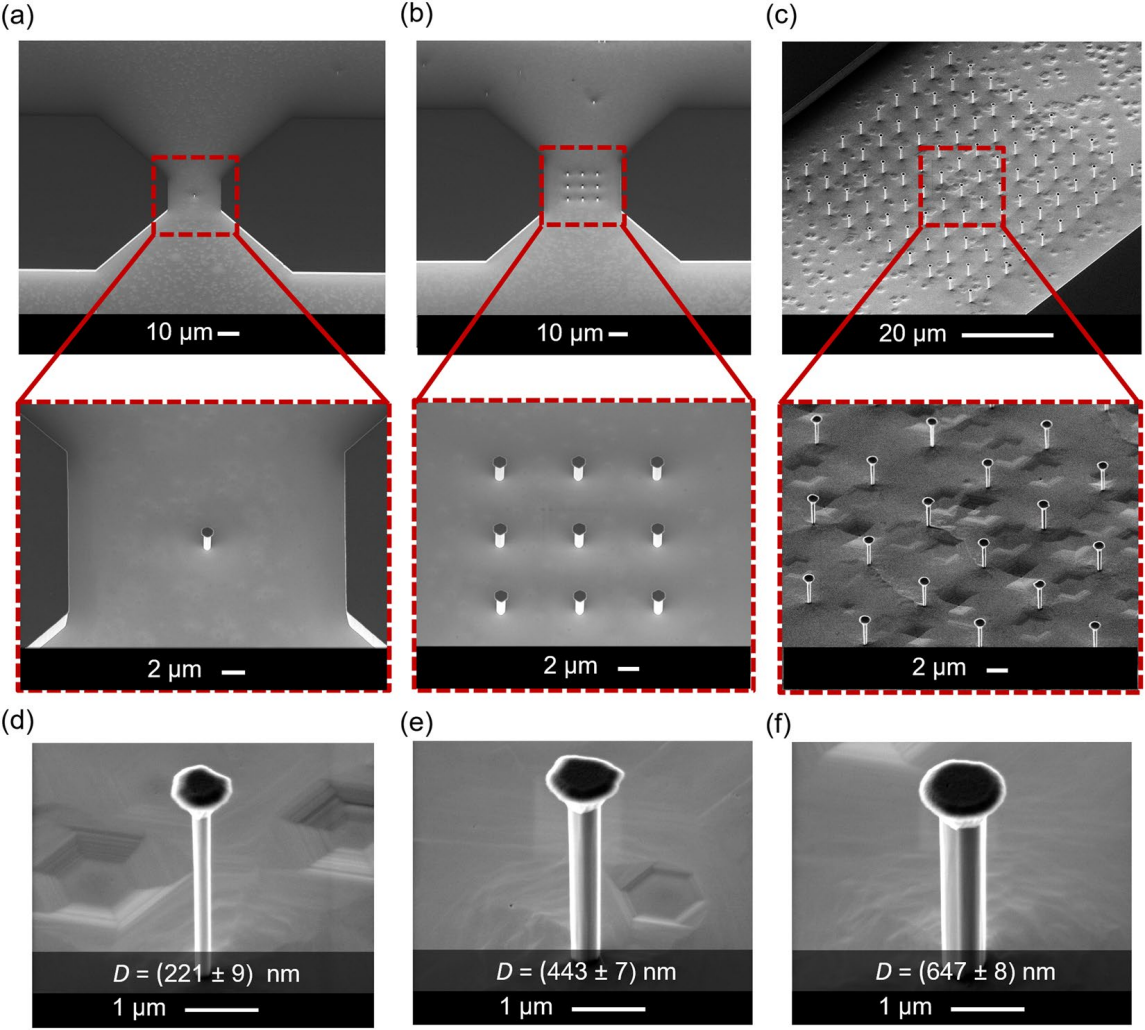
Bird-view SEM images of vertically aligned *n-p-n* GaN nanowire (NW) arrays after ICP-DRIE and wet chemical etching with NW numbers of (**a**) 1, (**b**) 9, and (**c**) 100. Varied wire diameters (*D*) of (**d**) (221 ± 9) nm, (**e**) (443 ± 7) nm, and (f) (647 ± 8) nm were obtained originating from different pattern sizes of the used Cr etching mask.

To investigate the scaling behavior of the integrated FETs, devices containing different numbers of NWs were fabricated, i.e., 1, 9, and 100 NWs (Fig. 2a–c). However, for the sake of simplicity, in the next result discussions, the three FET wire diameters of (221 ± 9) nm, (443 ± 7) nm, and (647 ± 8) nm (Fig. 2d–f) are written as 220 nm, 440 nm, and 640 nm, respectively. The formation of smooth sidewalls on vertical *n-p-n* GaN NWs after anisotropic wet chemical etching is explained in detail elsewhere^13,30,39^. Additionally, it is worthy to mention that the high quality of the etched NW sidewalls is important for the device performance^14^.

**Figure 3:**
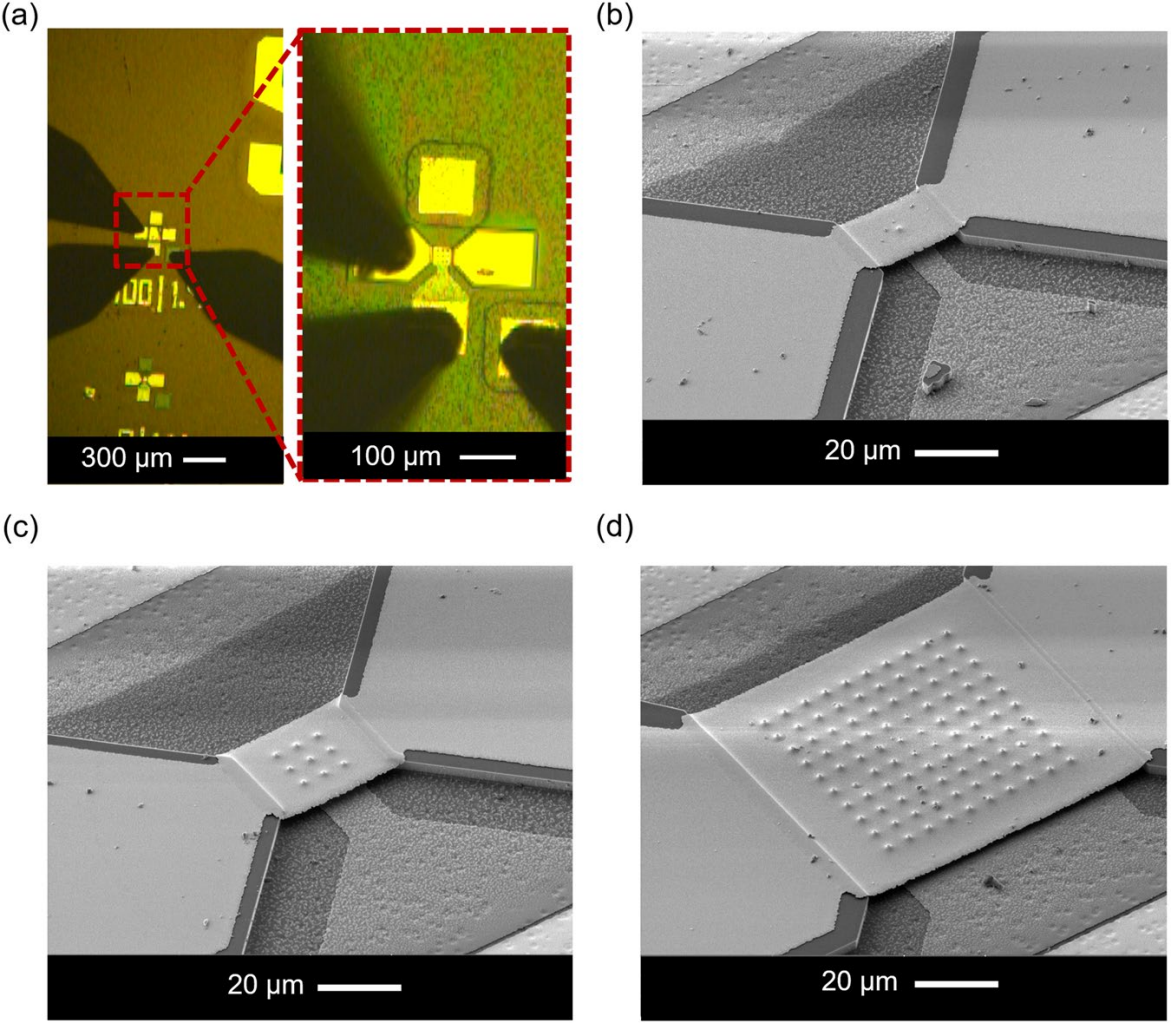
(**a**) Optical micrograph of a GaN FET device under test using a source meter unit (SMU) and probing tips. SEM images of vertical *n-p-n* GaN NW FETs consisting of (**b**) 1, (**c**) 9, and (**d**) 100 NWs.

Figure 3a shows the tested sample containing 1, 9, and 100 NW FETs connected with three probing tips to a source meter unit (SMU). The benefit of using two GaN mesa structures for drain contacts at the both sides of the NW field is the enhanced mechanical support during SMU probe testing or wire bonding process compared to devices with only photoresist or other soft materials underneath the metal pads^28,40,41^. It should be noted that no additional mask is required during the processing, as the drain pad was formed in line with the first lithography step for creating the NWs at the beginning of device fabrication. Consequently, the pad has the same height as the NWs. Figure 3b–d show the SEM images of fabricated vertical *n-p-n* GaN NW FETs with 1, 9, and 100 NWs including their top drain bridging contacts.

**Figure 4:**
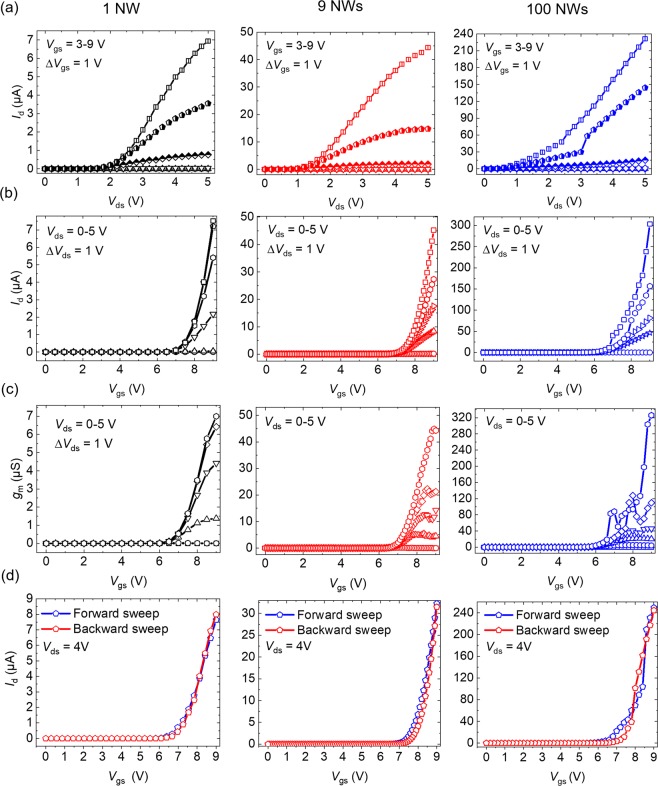
(**a**) Output characteristic, (**b**) transfer characteristic, (**c**) transconductance (*g*_m_), and (**d**) dual sweep mode transfer characteristic (sweep rate of 0.8–1 V/s) of 1, 9, and 100 *n-p-n* vertical GaN NW FETs with diameter of 440 nm at *V*_ds_ = 4, respectively. All measurements were done in normal ambient condition (room temperature) for at least five sweep cycles on each FET device.

Figure 4a presents the output characteristics of vertical *n-p-n* GaN NW FETs with a diameter of 440 nm and different numbers of 1, 9, and 100 NWs under the gate-source bias (*V*_gs_) ranging from 3 to 9 V. For each FET type, five different devices were measured on a single 2-inch epi-GaN wafer. The extracted maximum drain current (*I*_d,max_) at *V*_gs_ = 9 V demonstrates the linearity in respect to the number of NWs as shown in Table 1. The *I*_d,max_ and *I*_on_/*I*_off_ of each GaN NW FET scale up with the increasing number of NWs. Thus, it has proven the design suitability for massive parallelization. However, it should be noted that device processing imperfection can provide a deteriorated linearity of the devices, e.g., in the case of device scaling from 9 to 100 NW FETs, where their measured *I*_d,max_ are (33.5 ± 9.9) µA and (230.7 ± 59.4) µA, respectively. The normalized *I*_d,max_ (i.e., *I*_d,max_norm_) shows similar amounts of current density for FETs with different gate widths (*W*) and NW numbers, although the values are slightly varied from (1.7 ± 0.4) to (2.6 ± 0.9) µA/µm. The *W* is defined as the circumference of the single GaN NW, which has values of 0.69 µm, 1.38 µm, 2.01 µm for FETs with NW diameters of 220 nm, 440 nm, 640 nm, respectively.

**Table 1:** DC characteristic results of vertical *n-p-n* GaN NW FETs (average value) with NW numbers of 1, 9, and 100 and a diameter of 440 nm.

Number of NW (#)	*V*_th_ (V)	*I*_d,max_ (µA)	*I*_d,max_norm_ (µA/µm)	*I*_on_/*I*_off_	*I*_on_/*I*_off_norm_
1	(6.4 ± 0.2)	(3.2 ± 2.1)	(2.3 ± 1.5)	10^5^	10^5^
9	(6.6 ± 0.3)	(33.5 ± 9.9)	(2.6 ± 0.9)	10^6^	10^5^
100	(6.4 ± 0.5)	(230.7 ± 59.4)	(1.7 ± 0.4)	10^7^	10^5^

Those values are obtained by simplifying the cross-sectional geometry of the wire from hexagonal to circular shapes, as the wire dimension is considerably small. The *I*_d,max_norm_ can be calculated as follows:

1 $${I}_{{\rm{d}},{\rm{\max }}\_{\rm{norm}}}=\frac{{I}_{{\rm{d}},{\rm{\max }}}}{W\times N}=\frac{{I}_{{\rm{d}},{\rm{\max }}}}{\pi \times D\times N}\,$$

where *D* and *N* are diameter and number of the NWs, respectively. A similar method is used to obtain the normalized transconductance (*g*_m,max_norm_), i.e., by dividing the maximum transconductance (*g*_m,max_) with (*W* × *N*).

Regardless of the rectifying I-V behavior in lower *V*_ds_ field and the required contact optimization, the devices can still function in an enhancement mode (E-mode) operation. To improve the Ohmic contact, several solutions are suggested and will be attempted in future, such as usage of other metal materials (e.g., Ti/Al, Ti/Al/Ni/Au, or Ti/Cr/Au) with optimized thickness and annealing processes^42–45^.

The transfer characteristic is shown in Fig. 4b, plotting *I*_d_ as a function of *V*_gs_. The average *V*_th_ values determined by a linear extrapolation of the *g*_m_ (Fig. 4c) were (6.4 ± 0.2) V, (6.6 ± 0.3) V, (6.4 ± 0.5) V for the 1, 9, and 100 NW FETs, respectively, which are almost three times higher than the previously reported MOSFETs with c-axis GaN NWs^13^.

Compared to the massively parallel vertical Si NW FETs fabricated using e-beam lithography, these GaN NW FETs were believed to provide more stable process, regardless of the required engineering of the interface between the semiconductor and dielectric materials^26,46,47^. The ON-/OFF current ratios (*I*_on_/*I*_off_) of up to 10^7^, 10^6^, and 10^5^ were achieved for 100, 9, and 1 NW FETs, respectively (see SI Fig. S1), through the logarithmic *I*_d_-*V*_gs_ curves. Meanwhile, the normalized *I*_on_/*I*_off_ (i.e., *I*_on_/*I*_off_norm_), which is defined as *I*_on_/*I*_off_ per number of NWs, is found to be similar at values of 10^5^ for 1, 9, and 100 NW FETs (see Table 1). Those measured two parameters (*I*_on_/*I*_off_ and *I*_on_/*I*_off_norm_) have demonstrated the scalability and fabrication quality of the *n-p-n* GaN NW FETs, respectively. The higher *I*_on_/*I*_off_ on 100 NW FETs is reasonable since the *I*_d,max_ is proportional to number of NWs, in which for this NW FET the value is larger than those on 1 and 9 NW FETs, as depicted in Fig. 4b. The *g*_m,max_norm_ values (Fig. 4c) were measured as 6.3 mS/mm, 4.1 mS/mm, and 4.7 mS/mm for the 1, 9, and 100 NW FETs, respectively. The oxide capacitances in these FETs are around 4.7 pF, based on capacitive-voltage (C-V) measurements (see SI Fig. S2). Several solutions can be proposed to enhance the *g*_m_, including by decreasing *L* and increasing the *W* (i.e., shorter gate channel and larger NW diameter), as well as choosing the proper channel doping concentration design with the purpose of further increasing the electron mobility (*µ*) in *p*-channel to lower the scattering rate^48^. Review on vertical 3D GaN NW FETs including their important parameters to evaluate the device performances has been recently published elsewhere^25^.

From Fig. 4d, a gate memory effect or typical hysteresis caused by adsorbates, mobile ions, or interface/oxide traps is not visible in the fabricated devices, which demonstrates a good quality of the used dielectric material (i.e., higher electron affinity and particle density compared to SiO_2_)^13,14,49,50^. In addition, no obvious hysteresis was found on the 1 NW FETs with different diameters and on the 1, 9, 100 NW FETs with diameter of 220 nm (see SI Fig. S3). It should be noted that our previous NW FET devices using SiO_2_ gate dielectrics have large gate hysteresis during up-/down- current sweep^13^.

**Figure 5:**
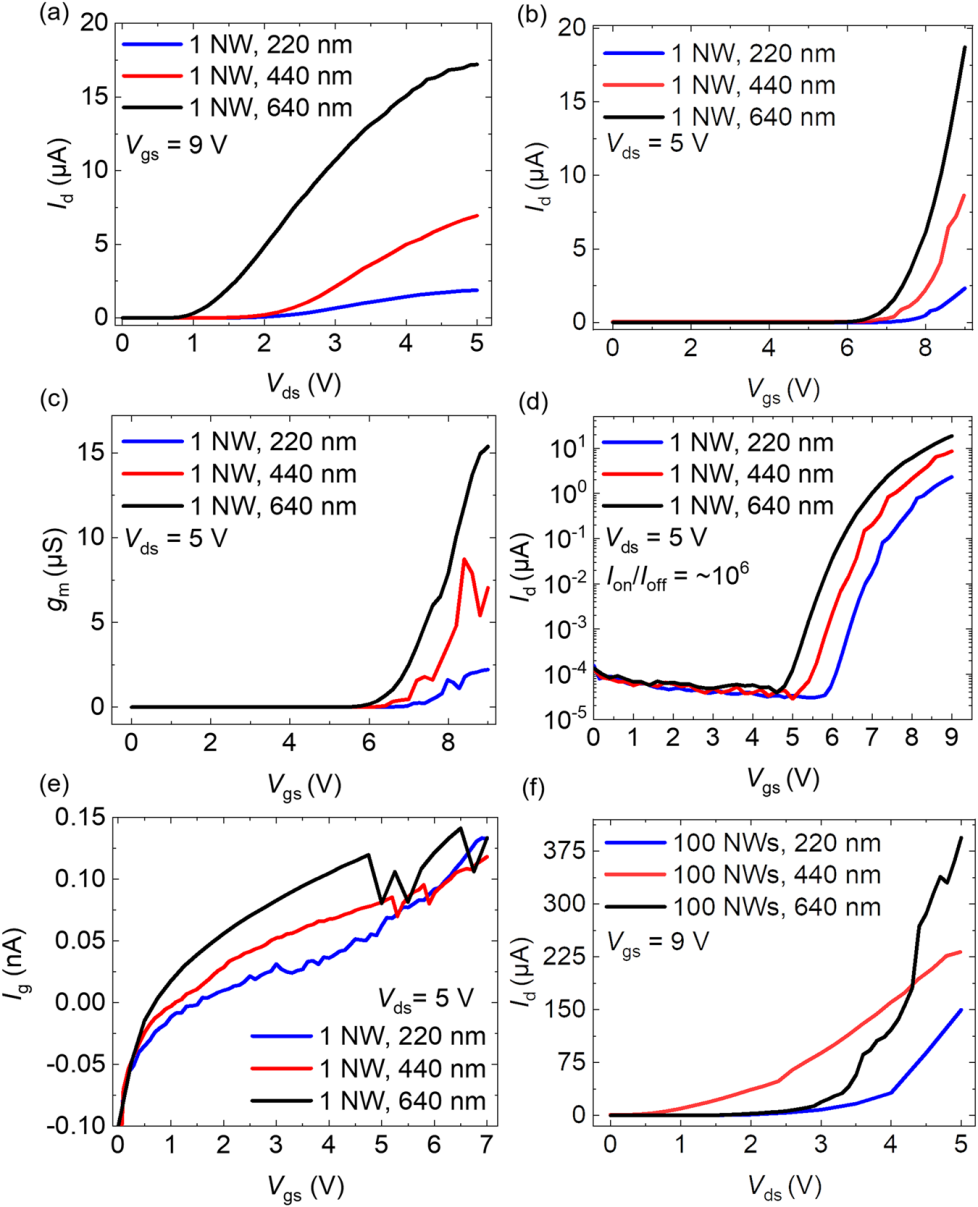
(**a**) Output characteristic, (**b**) transfer characteristic, (**c**) transconductance characteristic, (**d**) *I*_on_/*I*_off_ characteristic, (**e**) gate leakage characteristic of 1 NW FETs, and (**f**) output characteristic of 100 NW FETs with three different diameters (i.e., 220 nm, 440 nm, and 640 nm).

Figure 5a shows the output characteristics of three different single NW FETs with varied diameters (i.e., 220 nm, 440 nm, and 640 nm). The average *I*_d,max_ values at *V*_gs_ = 9 V from the five conducted measurements exhibit a linear trend, for all diameters with the lowest value found for 220 nm transistor, while the current difference of 7.4 µA was measured between 440 nm and 640 nm transistors. The detailed DC characteristics of the investigated single NW FETs are listed in Table 2.

**Table 2:** DC characteristic results of single vertical *n-p-n* GaN NW FETs (average value) with three different diameters of 220 nm, 440 nm, and 640 nm.

Diameter of NW (nm)	*V*_th_ (V)	*I*_d,max_ (µA)	*I*_d,max_norm_ (µA/µm)
220	(6.6 ± 0.6)	(2.2 ± 0.8)	(3.2 ± 2.0)
440	(6.4 ± 0.2)	(3.2 ± 2.1)	(2.3 ± 1.5)
640	(6.3 ± 0.3)	(9.5 ± 4.1)	(4.5 ± 2.1)

The transfer characteristics of the FETs with three different NW diameters are shown in Fig. 5b with an average *V*_th_ = (6.4 ± 0.4) V at *V*_ds_ = 5 V. Again, in Fig. 5c, *g*_m,max_norm_ values were inspected to be 8.8 mS/mm, 6.3 mS/mm, and 7.7 mS/mm for 220 nm, 440 nm, and 640 nm, respectively, with an *I*_on_/*I*_off_ of up to 10^6^ (Fig. 5d). The drain leakage (*I*_d,leak_) below the *V*_th_ was as low as ~10 pA (Fig. 5d) similar to the noise level of the measurement setup (1–10 pA), while the gate dielectric leakage current (*I*_g,leak_) was observed to be as low as ~0.07 nA/NW at *V*_ds_ = 5 V and *V*_gs_ = 6 V for those three different FETs (Fig. 5e). It proves that the high-*k* dielectrics, denser particle, and higher electron affinity of Al_2_O_3_ ultrathin void-free layer deposited by ALD have excellent properties on suppressing the *I*_d,leak_ and *I*_g,leak_ in our transistors^45^. Compared to single NW FETs, the output characteristic performances of 100 NW FETs have more linearity in case of different wire diameters (Fig. 5f).

**Figure 6:**
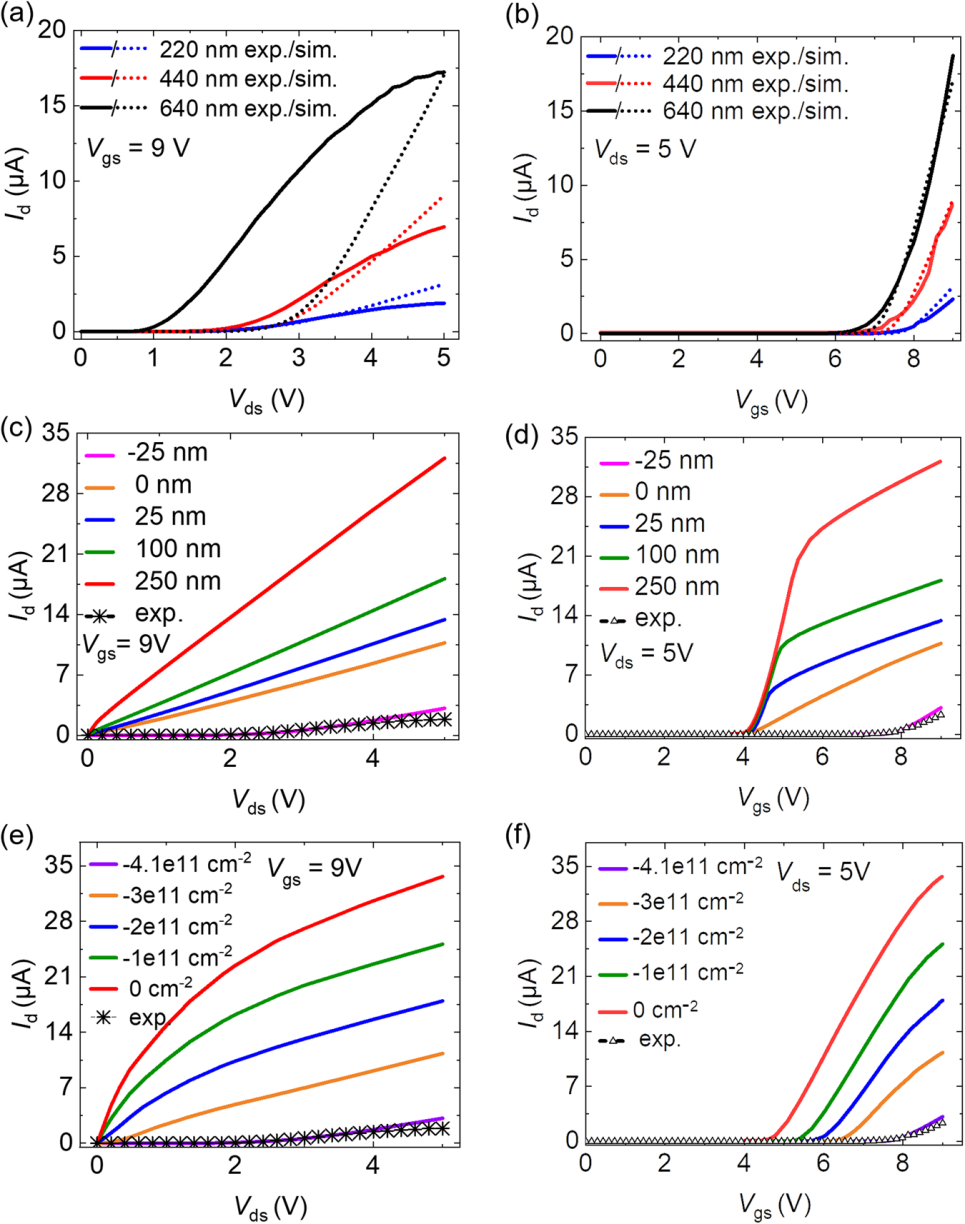
Experimental (exp.) and simulation (sim.) results showing (**a**) output and (**b**) transfer characteristics for single NW FETs of different diameters. (**c**) Simulated output and (**d**) transfer characteristics of different *L*_ext_ on single NW FETs with diameter of 220 nm and surface charge *Q*_sc_ = −(3.9 ± 0.2) × 10^11^ cm^−2^. The exp. data are used for comparison, which fit well with simulated results of FET with *L*_ext_ of −25nm. (**e**) Simulated output and (**f**) transfer characteristics of different *Q*_sc_ on NW sidewall in case of single NW FETs with diameter of 220 nm and *L*_ext_ = −(21 ± 4) nm.

To understand the phenomena of lower *I*_d,max_ and higher *V*_th_ obtained from the developed devices, the effects of gate length (*L*) and surface charge (*Q*_sf_) have been further studied by numerical simulations. The value of the gate length extension (*L*_ext_) describes the extension of the gate electrode beyond the *p*-channel region and results from the difference between the gate length *L* and the vertical position of the drain side *p-n* junction. The gate length is critical and should be kept as short as possible to limit the electric field in the drift region. A negative *Q*_sf_ on the nonpolar sidewalls of GaN NWs has been observed in experiments^51^. Its primary effect is to increase the threshold voltage of the transfer characteristics. Calibrating the simulations with the transfer characteristics yields the *Q*_sc_ = −(3.9 ± 0.2) × 10^11^ cm^−2^ and *L*_ext_ = −(21 ± 4) nm for all NW diameters. The gate electrode ends in the *p*-channel near the junction to the drift region (SI Fig. S5a,b calibrated). Figure 6b illustrates a very good agreement of simulation and experiment, which means some parameters in experiment in case of three different diameters of 1 NW FETs should be correct and can be explained by these conducted simulations.

The simulated output characteristics in Fig. 6a show relatively good agreement with the experimental results for the 220 nm and 440 nm NW FETs. However, a rather large difference of the turn-on voltage in the output characteristics of the 640 nm NW device suggests that the gate length value owned by the fabricated FET is probably higher, which is subject to the device processing. This will result in shorter channel length and consequently lower turn-on voltage (*I*_d_ − *V*_ds_), in comparison to that from simulation. Furthermore, the simulations in Fig. 6c,d demonstrate that the *I*_d_-*V*_ds_ and *I*_d_-*V*_gs_ curves are very sensitive to *L*_ext_. Due to deposition shading and the hexagonal wire shape, the gate metal length varies on the perimeter as depicted by the SEM image of a single wire in Fig. 1c. The variation increases with the diameter explaining the large deviation of the output characteristics for the 640 nm NW FET in Fig. 2a.

Even though the turn-on in the output characteristic may be also caused by imperfect Ohmic source or drain contacts, the simulations suggest that the high threshold and low transconductance can be attributed to the dimensions of the gate electrode. Moreover, surface charging effect has occurred on all FETs, which most probably originates from the impaired quality of the Al_2_O_3_ gate dielectric layer that has direct contact to GaN NW sidewalls. Nevertheless, all these phenomena need to be further investigated more deeply to understand the surface physics of the used materials from the realized FET devices.

Figure 6c,d illustrate the effects of the gate length on the output and transfer characteristics for the calibrated surface charge on 220 nm GaN NW FETs. With decreasing gate length, the resistivity increases and the transconductance decreases because the electron depletion in the drift region cannot be lifted by the gate electric field. For a very short gate ending in the *p*-channel region, the gate electric field does not screen the potential barrier at the junction to drift region anymore. Thus, a turn-on voltage in the output characteristics occurs. The negative surface charge does not only increase the threshold voltage as shown in Fig. 6f, but also contributes to the depletion of the drift region, which can be seen from the increase of resistivity in Fig. 6e. With increasing negative surface charge, the junction depletion region extends more into the drift region that contributes to the turn-on voltage in the output characteristics. It is remarkable that for a short gate configuration, the electric field in the drift region near the junction to the channel is larger than the field near the drain region, which exhibits a high resistivity. However, for a long gate architecture, this effect is not found (see SI Fig. S5a,b).

The measured BVs of the 1, 9, and 100 NW FETs were verified from 30–90 V at *V*_gs_ = 0 V as shown in SI Fig. S4. Theoretically, the BV is determined by the length and doping concentration of the drift region. In these devices, the space between drain and gate channel is ~1 µm, which is much shorter than our previous device having BVs of 54–92 V^13^. However, for improving the device design and performance, several strategies can be proposed for the next transistor generations, e.g., decreasing the Si-doping concentration in the drift region, integrating vertical field-plate structure, and employing longer drift region. The low doped and longer space drift region will reduce the local electric field and thus the BV can be increased^20,29,52^.

In conclusion, E-mode vertical *n-p-n* GaN NW FETs with different properties (i.e., NW numbers and diameters) have been fabricated and demonstrated, proving the feasibility for upscaling the vertical transistors by optimizing the dielectric materials and device designs. The smooth vertical sidewalls of the *n-p-n* NWs have been realized by a hybrid top-down approach involving dry and wet chemical etching techniques. Regardless of the required device processing optimization, the fabricated FETs have exhibited *V*_th_ of up to (6.6 ± 0.3) V and shown an upscaling *I*_d,max_ behavior with increasing NW quantity from 1 to 100 and NW diameter from 220 nm to 640 nm. Through appropriate dielectric passivation material deposited on the entire NWs, the gate hysteresis effect observed in earlier devices could be suppressed to nearly zero. To enhance *I*_d,max_, the NW diameter can be further controlled (larger *W*) and the NW number can be employed (*N* > 100 NWs). These results grant a very promising future design for massively parallel GaN vertical transistors to be used in logic circuitry and metrological applications (i.e., to be extended for single-electron transistors (SETs) providing alternatives for parallel planar GaAs SETs^53,54^).

## Methods

### GaN NW array preparation

The NW arrays were fabricated out of epitaxial GaN layers grown by metalorganic vapor-phase epitaxy (MOVPE) on sapphire substrates in a top-down hybrid etching approach. The epitaxial wafer has an *n-p-n* layer stack structure (Fig. 1b). Afterwards, the wafers were patterned by photolithography and lift-off technique with Cr as dry etching mask. Hexagonal-cone GaN nanostructure arrays were obtained by ICP-DRIE with SF_6_ and H_2_ gases. Furthermore, KOH-based wet chemical etching was carried out to remove the plasma-induced surface damage of the nanostructures and to shrink the diameter of the hexagonal structures realizing smooth vertical sidewalls. The NW diameters were varied as defined during photolithography to be (221 ± 9) nm, (443 ± 7) nm, and (647 ± 8) nm after ICP-DRIE and wet chemical etching, while the NW height was kept the same at around 3 µm^20^.

### Transistor fabrication

In 3D device processing of vertical GaN NW FETs, highly *n*-doped GaN was used on top layer of the NWs to improve the quality of the Ohmic contacts. The Mg acceptors in the gate channel (*p*-channel) of *n-p-n* GaN NWs were activated by rapid thermal annealing (RTA) at 950 °C for 30 s and subsequently at 600 °C for 5 min. Furthermore, thermal ALD technique using trimethylaluminium and water was employed to form a ~25 nm thick Al_2_O_3_ gate dielectric layer. To improve the passivation between the GaN layer and the gate metal, a 200 nm thick SiO_x_ was deposited using e-beam evaporation. The wrap around Cr gate (Fig. 1c) was deposited via tilted e-beam evaporation, using the shadowing effect from the mushroom-like NW shape to prevent any deposition on the upper part of the NWs. Thus, it covered the 0.4 µm *p*-channel area on the GaN epi-layer (Fig. 1b) and is located 1 µm below the top mushroom head of NWs (see inset Fig. 1a). The *W* is the circumference of the NWs with diameters of 220 nm, 440 nm, and 640 nm. For supporting the drain contact, the space between the GaN NWs was filled with photoresist with short UV exposure and cured at around 250 °C for 30 min (Fig. 1d). Next, to form an Ohmic contact, a Cr/Au (80/200 nm) layer stack was deposited (see Fig. 1e). Afterwards, this sacrificial polymer could be removed if necessary resulting in top drain bridging contact.

### DC characterization

The output and transfer characteristics of FETs were measured by an SMU Keithley 4200-semiconductor characterization system (SCS) at room temperature and under parasitic electric charge protection. Three point-like gold probes with coaxial cables probes were used to enhance the electric transfer between the device and the SMU. All *V*_th_ and *I*_d,max_ values were conducted from five measurements of the same FETs. Hence, the average values and their standard deviations were finally taken.

### Simulation Model

The numerical simulations have been carried out with Sentaurus Device using hydrodynamic carrier transport for electrons and drift-diffusion transport for holes. The lattice temperature is 300K for all simulations. The hexagonal NW was approximated by a cylindrical geometry and simulated on a 2D radial cross section using azimuthal expansion. The gate isolation material (Al_2_O_3_) has been assumed to be crystalline with a permittivity *ε* = 10.2. A calibration of the gate capacitance was not possible due to the large contribution of the contact capacitance in the experiment (SI Fig. 2). The carrier mobility model incorporates high field saturation of the velocity as well doping dependence^55^. The acceptor doping of the channel region and the donor doping of the drift region are *N*_A_ = 2 × 10^18^ cm^−3^ and *N*_D,drift_ = 5 × 10^16^ cm^−3^, respectively, and calibrated with experimental results in Fig. 5a,b.

## Supplementary information

Supplementary Information

## Data Availability

All data generated or analyzed during this study are included in this published article (and its Supplementary Information (SI) files).
