# Supplementary material for: Top-down GaN nanowire transistors with nearly zero gate hysteresis for parallel vertical electronics
Source: Sci Rep. 2019 Jul 16;9:10301. doi: 10.1038/s41598-019-46186-9 (PMC6635513; doi:10.1038/s41598-019-46186-9)
Supplement: Supplementary file 1 — Supplementary Information [file 41598_2019_46186_MOESM1_ESM.pdf]

Supplementary Information to:

Top-down GaN nanowire transistors with nearly zero gate hysteresis for parallel vertical electronics

Muhammad Fahlesa Fatahilah[1,2,*], Feng Yu[1,2], Klaas Strempel[1,2], Friedhard Römer[4], Dario Maradan[3], Matteo Meneghini[5], Andrey Bakin[1,2], Frank Hohls[3], Hans Werner Schumacher[3], Bernd Witzigmann[4], Andreas Waag[1,2], Hutomo Suryo Wasisto[1,2,*]

1 Institute of Semiconductor Technology (IHT), Technische Universität Braunschweig, Hans-Sommer-Straße 66, D-38106 Braunschweig, Germany
2 Laboratory for Emerging Nanometrology (LENA), Technische Universität Braunschweig, Langer Kamp 6, D-38106 Braunschweig, Germany
3 Physikalisch-Technische Bundesanstalt (PTB), Bundesallee 100, D-38116 Braunschweig, Germany
4 Computational Electronics and Photonics (CEP), University of Kassel, Wilhelmshöher Allee 71, D-34121 Kassel, Germany
5 Department of Information Engineering, University of Padua, 35131 Padua, Italy

* Corresponding authors; Tel.: +49 531 391 3167; Fax: +49 531 391 5844
E-mail address: m.fatahilah@tu-braunschweig.de (M.F. Fatahilah), h.wasisto@tu-braunschweig.de (H.S. Wasisto)

1. I_{on}/I_{off} ratio

The I_{on}/I_{off} for different numbers of NWs with similar diameter shown in Fig. S1 was taken by logarithmic of transfer characteristics.

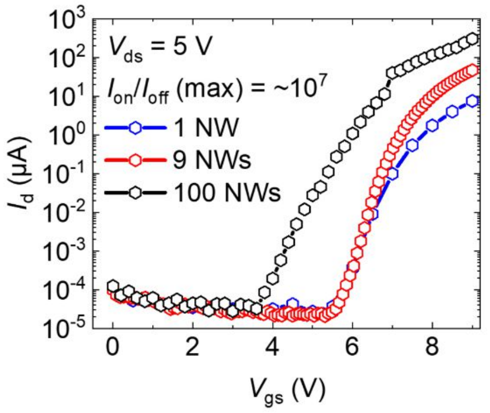

Figure S1. I_{on}/I_{off} measurement (I_d- V_{gs} logarithmic) curves of GaN NW FETs with a diameter of 440 nm and different numbers of 1, 9, and 100 NWs.

The ON-/OFF current ratio (I_{on}/I_{off}) increases with the higher number of integrated NWs. This is due to the fact that this ratio depends on the value of $I_{d,max}$ from the transfer characteristics, in which it will increase for the higher number of GaN NWs as shown in Table 1 (main article). From the logarithmic I_d- V_{gs} curves, the I_{on}/I_{off} values for 100, 9, and 1 NW FETs could reach up to 10^7, 10^6, and 10^5, respectively (see SI Fig. S1). Meanwhile, the normalized I_{on}/I_{off} (i.e., I_{on}/I_{off_norm}), which is defined as I_{on}/I_{off} per number of NWs, is found to be similar at values of ~10^5 for 1, 9, and 100 NW FETs (see Table 1). Those measured two parameters (I_{on}/I_{off} and I_{on}/I_{off_norm}) have demonstrated the scalability and fabrication quality of the *n-p-n* GaN NW FETs, respectively.

The V_{th} was slightly different due to effect of the logarithmic relation, but the real V_{th} as mentioned in main text was plotted as derivative relation yielding equation[1]:

$$g_m = \mu C_{ox} \frac{W}{L} (V_{gs} - V_{th}) \quad (1)$$

where W and L are the width and length or height of gate metal, μ is the carrier mobility, C_{ox} is the oxide capacitance. The oxide capacitances in these FETs are around 4.7 pF, based on capacitive-voltage (C-V) measurements, as shown in Fig. S2. The capacitance values in all FETs are similar to each other because of their arrangement in series connection (i.e., NW array architectures).

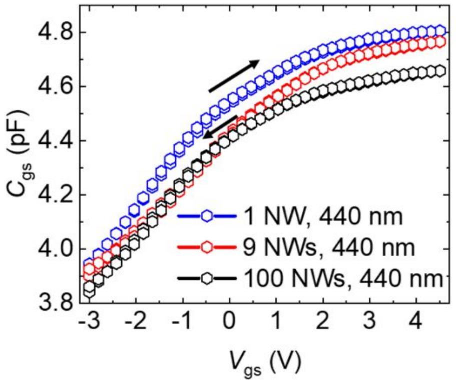

Figure S2. Dual sweep mode C-V measurement results of 440 nm NW FETs with different wire numbers.

2. Dual sweep mode transfer characteristics

In comparison with our previously developed *n-p-n* NW FETs using SiO_2 dielectric layers[2], which possess a gate voltage hysteresis of $\Delta V_{th} = 1.5$ V, Fig. S3 has shown a successful reduction of the hysteresis by employing the Al_2O_3 in these proposed NW FETs. The single NW FETs in Figs. S3 a,b,c with different diameters demonstrate very low measured gate hysteresis values ranging from $\Delta V_{th} = 0.2$ V to $\Delta V_{th} = 0.4$ V. Very low gate hysteresis was also measured for 1, 9, and 100 NWs FETs with diameter of 220 nm as shown in Figs. S3 d,e,f. However, without denying the very low hysteresis in 100 NW FETs with diameter of 220 nm (Fig. S3f), the V_{th} on this NW FETs is lower than those on other NW FETs, which can be attributed to device processing (i.e., variation of gate length affected by the mushroom head of GaN NWs during device processing and different condition of surface charges) as explained by simulation results in Fig. 6.

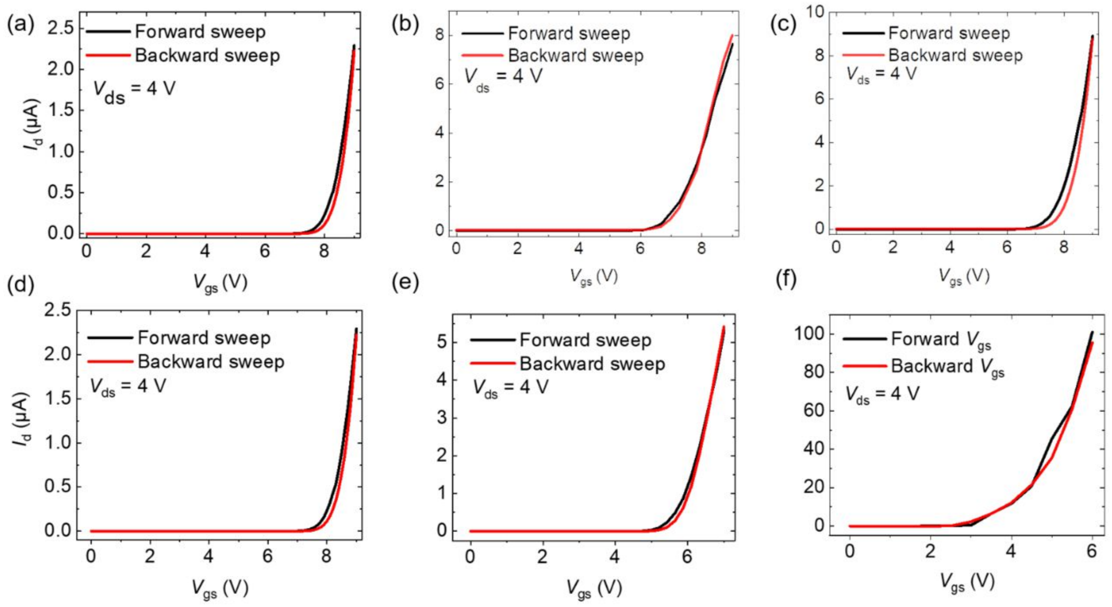

Figure S3. Dual sweep mode transfer characteristics (sweep rate of 0.8 - 1 V/s) of single NW FETs with diameters of (a) 220 nm, (b) 440 nm, and (c) 640 nm. (d) Dual sweep mode for single, (e) 9, and (f) 100 NW FETs with diameter of 220 nm. All measurements were done in normal ambient condition (room temperature) for at least five sweep cycles on each FET device.

3. Breakdown voltage

The breakdown voltages (BVs) have varied values from 30-90 V for all developed FETs, in which the largest BV was obtained by the 100 NW FET with a diameter of 220 nm (Fig. S4). The exact reason for this is still unclear. However, it can be attributed to inhomogeneity of drift region doping profile and location of gate Cr metal edge on NW FETs.

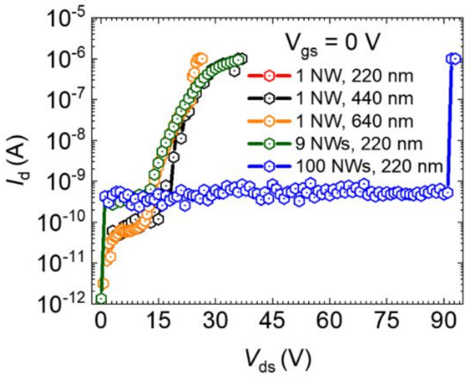

Figure S4. Breakdown voltages (BVs) of vertical *n-p-n* GaN NWs FETs with different NW numbers and diameters.

4. TCAD simulation

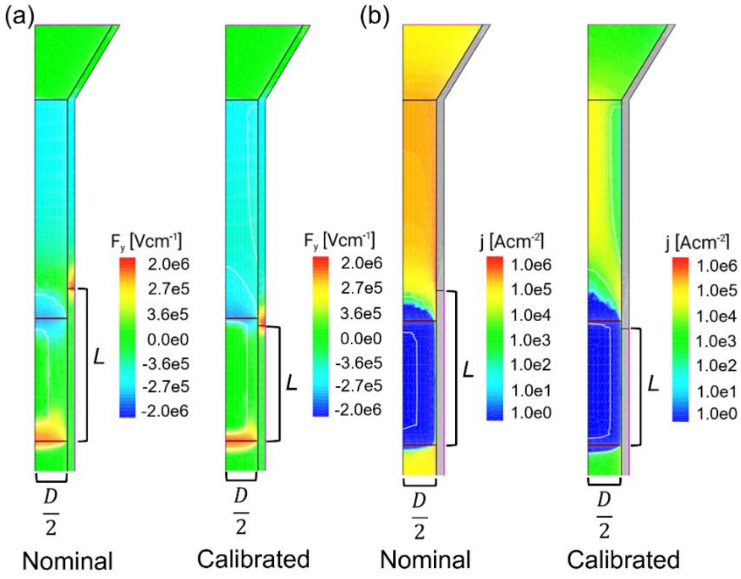

Figure S5. Effects of surface charging and gate extension on (a) electrical field F_y (V/cm) and (b) current density j (A/cm^2) at the channel and the drift region. Simulations have been performed based on the original experimental parameters (i.e., nominal) and adapted simulation parameters (i.e., calibrated) on single GaN NW FET with diameter of 220 nm. The L and D are gate length and wire diameter, respectively.

From simulation results (Fig. S5), it has been demonstrated that the gate length (L) of the transistors measured in the experiments has a value of ~0.39 µm. Meanwhile, more investigation is still required to confirm the surface charge (Q_{sc}) effect, which is most probably caused by the impaired quality of gate oxide (Al_2O_3) deposited by atomic layer deposition (ALD). Thus, several parameters used in ALD (e.g., deposition temperature and cycle) should be improved to reduce the Q_{sc} in Al_2O_3 having direct contact with GaN NW surfaces[3].

References

1. Cartwirght, K. V. Derivation of the Exact Transconductance of a FET without Calculus. *Technology Interface Journal* **10**, 7 (2009).
2. Yu, F. *et al.* Normally Off Vertical 3-D GaN Nanowire MOSFETs with Inverted p-GaN Channel. *IEEE Transactions on Electron Devices* **65**, 2439–2445 (2018).
3. Fitts, J. P., Shang, X., Flynn, G. W., Heinz, T. F. & Eisenthal, K. B. Electrostatic Surface Charge at Aqueous/α-Al2O3 Single-Crystal Interfaces as Probed by Optical Second-Harmonic Generation. *The Journal of Physical Chemistry B* **109**, 7981–7986 (2005).
